# Supplementary material for: How soap bubbles freeze
Source: Nat Commun. 2019 Jun 18;10:2531. doi: 10.1038/s41467-019-10021-6 (PMC6582157; doi:10.1038/s41467-019-10021-6)
Supplement: Supplementary file 1 — Supplementary Information [file 41467_2019_10021_MOESM1_ESM.pdf]

Supplementary Information

How soap bubbles freeze

S. Farzad Ahmadi,† Saurabh Nath,† Christian M. Kingett,† Pengtao Yue,§

and Jonathan B. Boreyko†,‡,*

†Department of Biomedical Engineering and Mechanics, Virginia Tech,
495 Old Turner Street, 222 Norris Hall, Blacksburg, VA 24061, USA
§ Department of Mathematics, Virginia Tech, Blacksburg, VA 2406, USA
‡Department of Mechanical Engineering, Virginia Tech, Blacksburg, VA 24061, USA.
*To whom correspondence should be addressed; E-mail: boreyko@vt.edu.

Supplementary Fig. 1

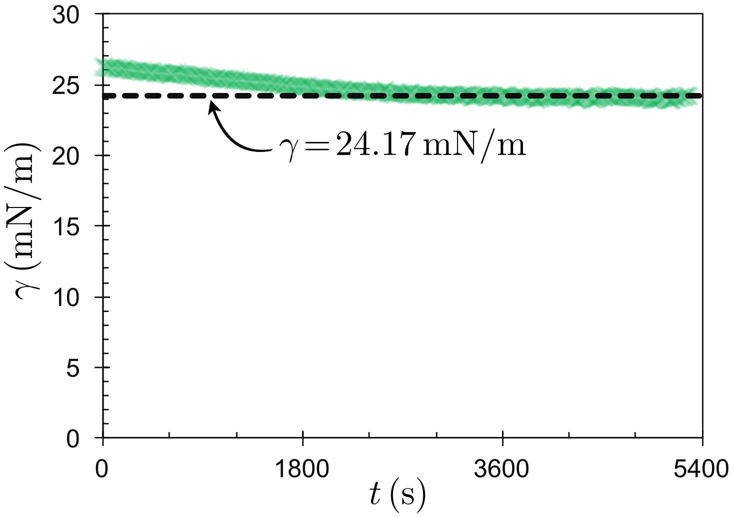

Supplementary Fig. 1: Surface tension measurements. The pendant drop method was used to measure the surface tension of the solution-air interface (ramé-hart, Model 590). The air temperature was $T_\infty = 21.3 \pm 0.7\,°\mathrm{C}$ with a relative humidity of $RH = 36\%$. The slight decay in the measured surface tension over time was a result of the presence of 1% surfactant (i.e. dish soap) in the solution. After about 40 min, the surface tension value remained constant at $\gamma = 24.17\,\mathrm{mN/m}$.

Supplementary Fig. 2

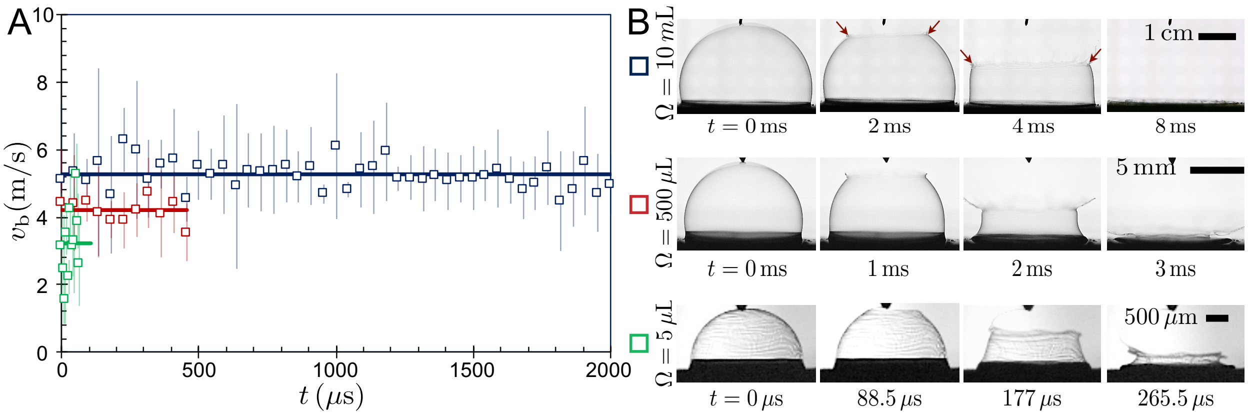

Supplementary Fig. 2: Bursting velocity of bubbles. (A) Velocity of the hole's rim moving along the arc of the bubble, measured intermittently up until the rim exhibited splashing. Data series correspond to interior volumes of: $\Omega = 5\,\mu$L (green squares), $\Omega = 500\,\mu$L (red), and $\Omega = 10\,$mL (blue). Error bars correspond to one standard deviation between three trials. The air and aluminum substrate were both at room temperature, $T_\infty \approx 22 \pm 1\,^\circ$C; the air had a relative humidity of $RH = 26\%$. Constant bursting velocities (solid lines) were found from the average of all the data points for a given bubble size. (B) High-speed image sequences of bursting bubbles used to measure the receding velocities of the liquid rims shown in (A). Bubbles were initially punctured with a sharp dry needle at their top. Red arrows in the first row of images show the evolving location of the rim.

Supplementary Fig. 3

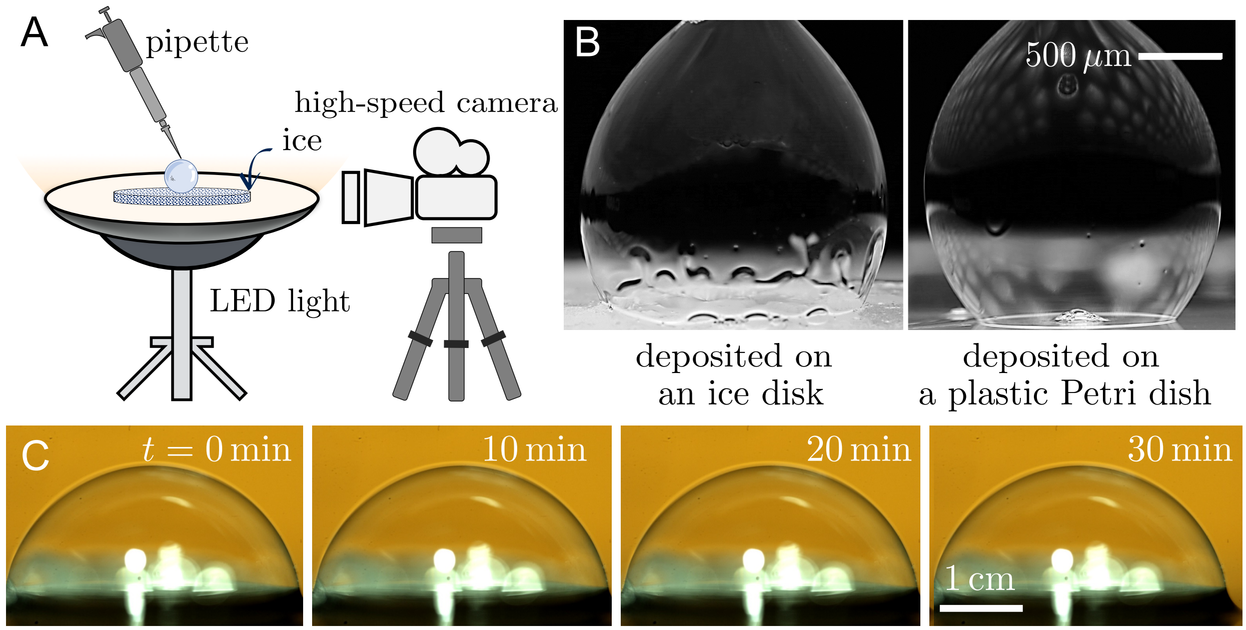

Supplementary Fig. 3: Experimental setup for the isothermal environment. (A) An ice disk and underlying light were placed on the floor of a walk-in freezer $T_\infty \approx -20\,^\circ$C. The freezing dynamics of deposited bubbles were then captured with a high-speed camera. (B) When the bubble was deposited on an ice disk, a strong Marangoni flow was observed during freezing (left image). In contrast, no Marangoni flow was observed when the bubble was deposited on a dry Petri dish (right image). This shows that the Marangoni flow is a direct result of the latent heat input by the freeze front. (C) When depositing the bubble on a dry silicon wafer in the freezer, there were no freezing events or Marangoni flow even after 30 min.

Supplementary Fig. 4

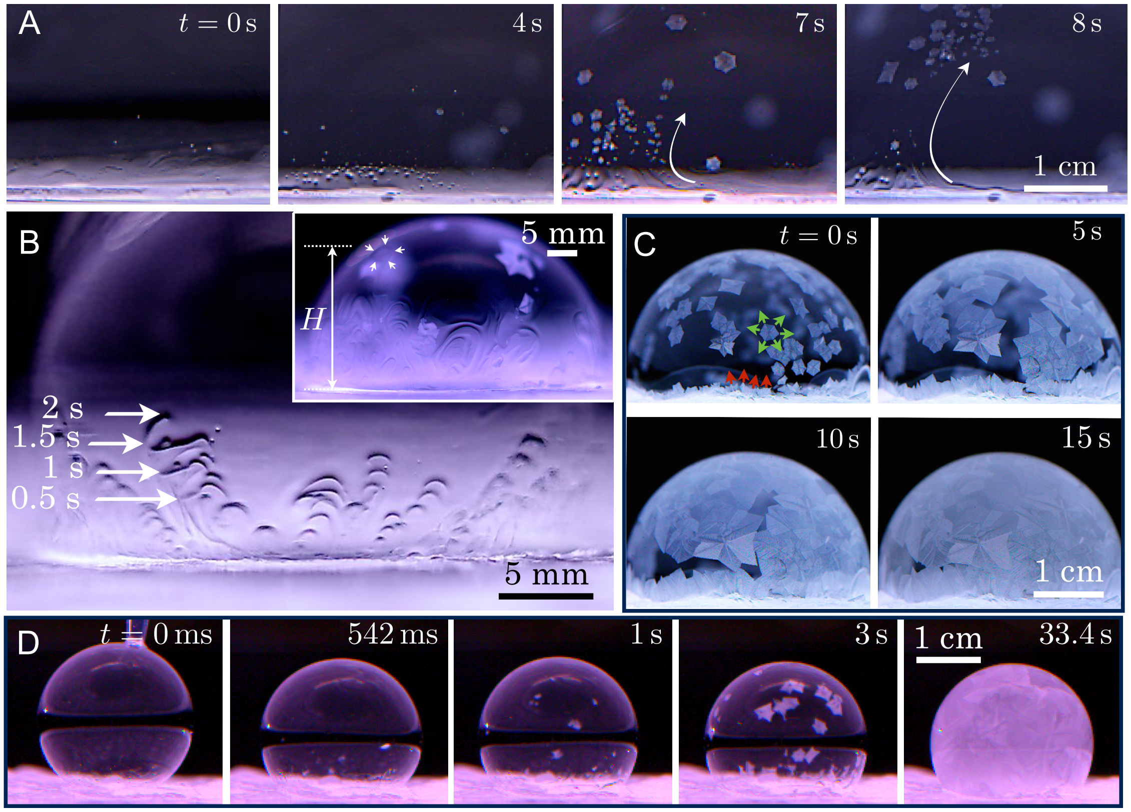

Supplementary Fig. 4: Marangoni flow for bubbles freezing in an isothermal environment. (A) Visualization of the bottom-up Marangoni flow caused by local heating at the freeze front. This flow removed ice crystals from the freeze front and carried them up the bubble (white arrows). The bubble was deposited on an ice disk in a walk-in freezer with $T_\mathrm{w} \approx T_\infty = -19 \pm 1\,^\circ\mathrm{C}$. (B) Time-lapse sequence of the Marangoni flow that resulted from depositing a bubble of volume $\Omega = 10\,\mathrm{mL}$ on an ice disk in the freezer ($T_\mathrm{w} \approx T_\infty = -19.6\,^\circ\mathrm{C}$). Time zero corresponds to when the bubble was first deposited onto the ice disk. Arrows show the location of the Marangoni flow. The inset shows the height (H) at which a thermal plume travels when a $\Omega = 10\,\mathrm{mL}$ bubble was deposited on an ice disk in a walk-in freezer with $T_\mathrm{w} \approx T_\infty = -19.1\,^\circ\mathrm{C}$. Arrows show the periphery of the thermal plume. (C) Bubbles froze from the cooperative growth of the bottom-up freeze front (red arrows) and the suspended ice particles (green arrows). (D) The transient time scale ($\sim 100\,\mathrm{ms}$) required for a $\Omega = 10\,\mathrm{mL}$ bubble to achieve the equilibrium angle is quite small relative to total freezing time scale ($\sim 10\,\mathrm{s}$). In other words, the ratio of spreading time to the freezing time is $\mathcal{O}(10^{-2})$.

Supplementary Fig. 5

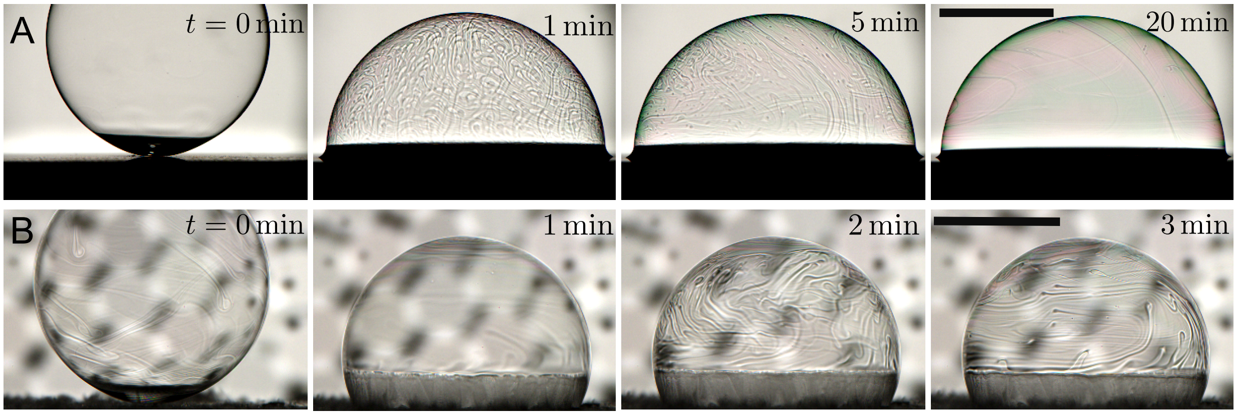

Supplementary Fig. 5: Marginal regeneration. (A) For a bubble deposited on a dry, room temperature substrate, plumes were continually generated through the ~10 min lifetime of the bubble due to marginal regeneration. In this case, the marginal regeneration is likely due to the mismatch in curvatures of the liquid meniscus wetting the surface versus the curvature of the bubble dome. (B) For a bubble deposited on a chilled substrate ($T_w = -20 \pm 1\,^\circ$C), marginal regeneration occurs only after the bubble reached to its partially frozen equilibrium (~ 100 s), most likely due to film drainage toward the frozen portion of the bubble. For both (A) and (B), experiments were conducted in a room with $T_\infty = 24.2 \pm 0.8\,^\circ$C and $RH = 25 \pm 8\%$. Scale bars represent 5 mm.

Supplementary Fig. 6

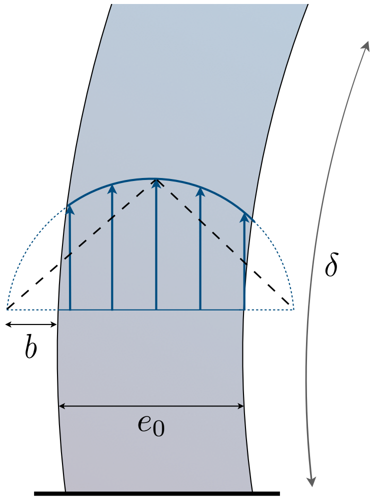

Supplementary Fig. 6: Schematic of flow inside the liquid film. Schematic showing the notations used in Eq. 2 of the main text where the surface tension gradient, $\Delta\gamma/\delta$, was balanced by viscous stress, $\eta V/(b+e_0/2)$. As is shown, δ is the length scale of the temperature gradient driving the flow and b is the slip length of the Poiseuille flow along the bubble's film. Blue arrows show the parabolic velocity profile while the black dashed lines show the constant velocity profile used in this study as an approximation.

Supplementary Fig. 7

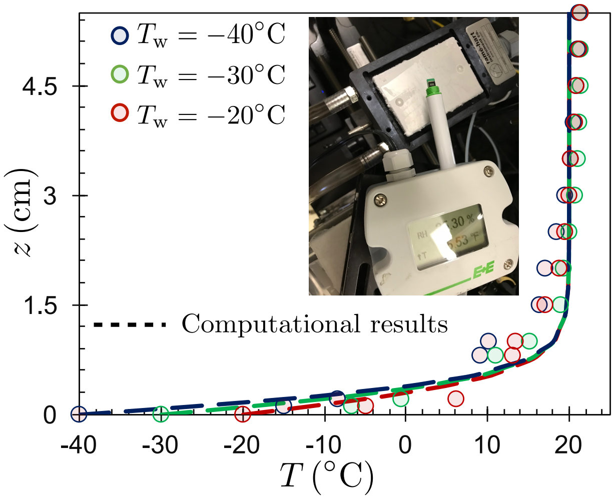

Supplementary Fig. 7: Measurements of the air temperature profile above the substrate. Without any bubbles on the substrate, the air temperature profile was measured as a function of the height above the Peltier stage. The surface temperature of the Peltier was either $T_w = -40\,°C$ (blue circles), $T_w = -30\,°C$ (green), and $T_w = -20\,°C$ (red). These experimental results were validated against the corresponding computational results obtained using COMSOL Multiphysics (dashed lines).

Supplementary Fig. 8

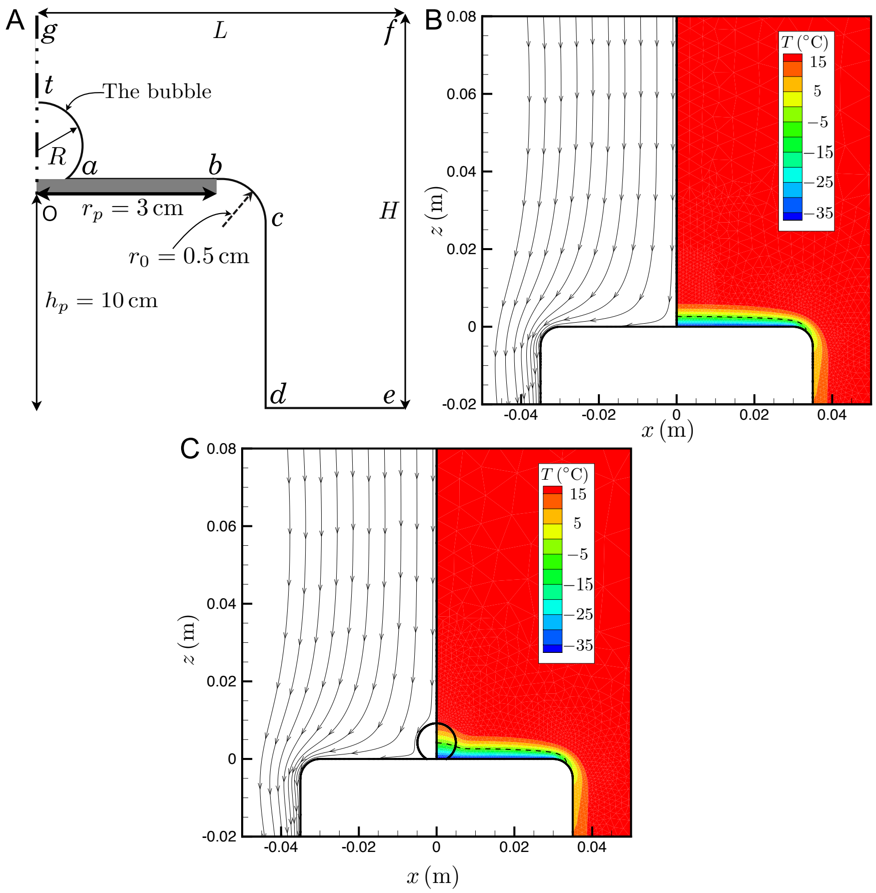

Supplementary Fig. 8: Simulation of the air temperature profile above the substrate. (A) Schematic of the computational domain defined in COMSOL for the the air temperature measurements. (B) Computational solution of Supplementary Eqs. 1 to 3 for a Peltier stage temperature of $T_\text{w} = -40\,^\circ\text{C}$ in the absence of a deposited bubble. The left half of this image depicts the velocity field due to natural convection, while the right half shows the resulting temperature field. (C) Computational solution with a $\Omega = 500\ \mu\text{L}$ bubble placed in the center of a Peltier stage set to $T_\text{w} = -40\,^\circ\text{C}$. The dashed line in (B) and (C) corresponds to $T = -6\ ^\circ\text{C}$.

Supplementary Fig. 9

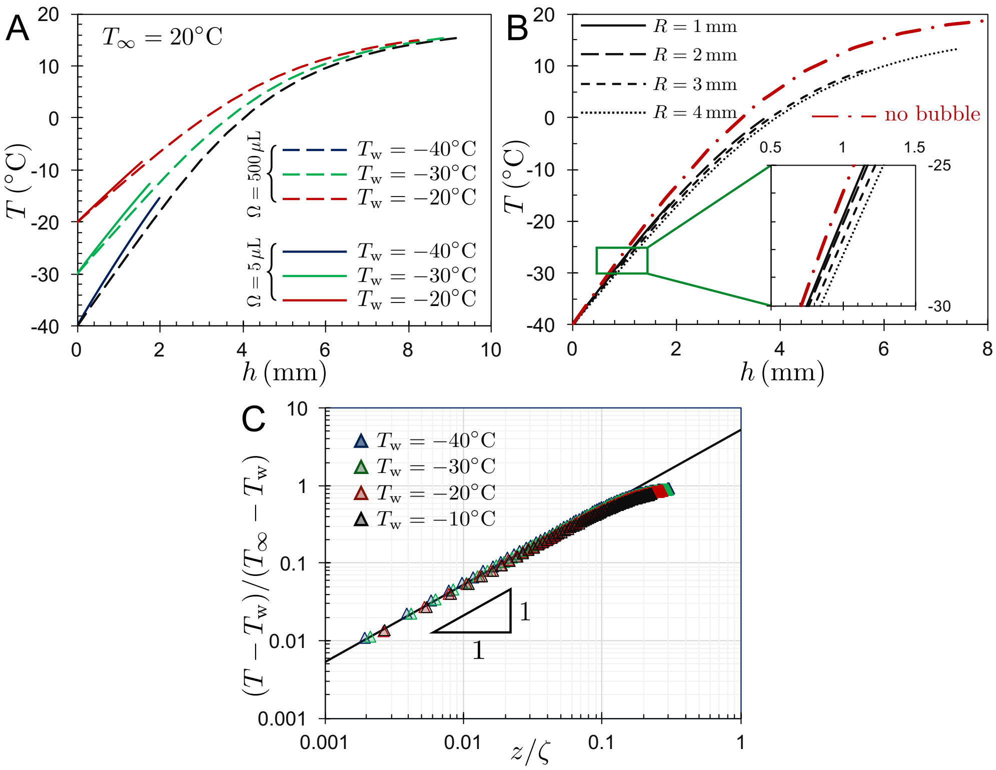

Supplementary Fig. 9: Numerical results of temperature fields for room temperature conditions. (A) Steady-state temperature profiles of bubbles (prior to freezing) as a function of the height above the chilled Peltier stage. The two different bubble volumes correspond to those used experimentally: $\Omega = 5\ \mu$L (solid lines) and $\Omega = 500\ \mu$L (dashed lines), while the Peltier was either $T_w = -20\,°$C (red), $T_w = -30\,°$C (green), or $T_w = -40\,°$C (blue). (B) Steady-state temperature profiles of bubbles, where the Peltier is now fixed at $T_w = -40\,°$C while the bubble size is more widely varied from $R = 1$ mm to $R = 4$ mm (or no bubble at all). (C) When the temperature is non-dimensionalized with respect to $T_\infty - T_w$ and plotted against z/ζ, the computational data collapses onto a single curve for $z/\zeta < 0.1$. In this regime, the air is primarily cooled by conduction ($\nabla^2 T = 0$, black line). For $z/\zeta > 0.1$, natural convection becomes increasingly important. For all of these simulations, the air temperature was kept at a constant value of $T_\infty = 20\,°$C while the contact angle of the bubble was $\psi_0 = 149.0°$ similar to that seen in the experiments.

Supplementary Fig. 10

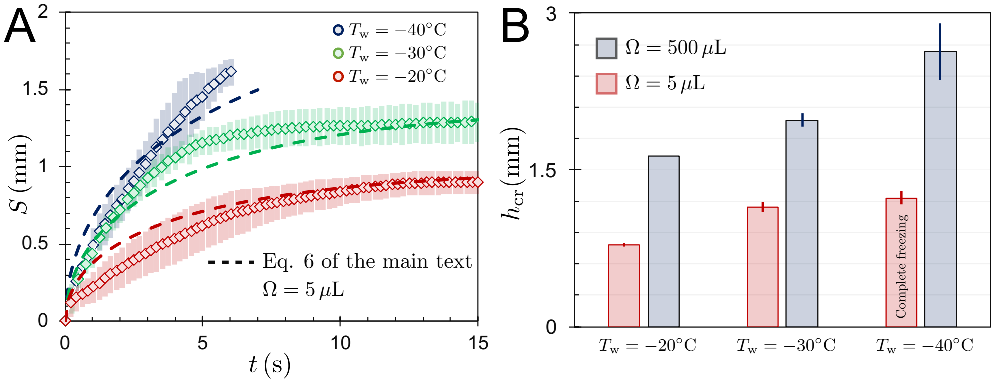

Supplementary Fig. 10: Freeze front dynamics in a room temperature environment. (A) Experimental measurements (data points) and theoretical model (dashed lines) of the frozen arc length (S) against time. This graph is specifically for $\Omega = 5\,\mu L$ bubbles, complementing Figure 4D in the main manuscript which focuses on $\Omega = 500\,\mu L$ bubbles. The maximal value of S represents a complete bubble freezing for a surface temperature of $T_w = -40\,°C$ and represents the halting of the freeze front for $T_w = -20\,°C$ and $T_w = -30\,°C$. The theoretical results correspond to Equation 6 from the main text, where $\beta_1 = 1$ and $\beta_2 = 30$. (B) The mean critical height from the substrate where the freeze front stopped for $\Omega = 5\,\mu L$ (red) and $\Omega = 500\,\mu L$ (blue) bubbles versus surface temperature. Error bars show one standard deviation between three trials. The average ambient conditions across all experiments were $T_\infty = 23.4 \pm 1.2\,°C$ and a relative humidity of $RH = 42 \pm 14\%$.

Supplementary Fig. 11

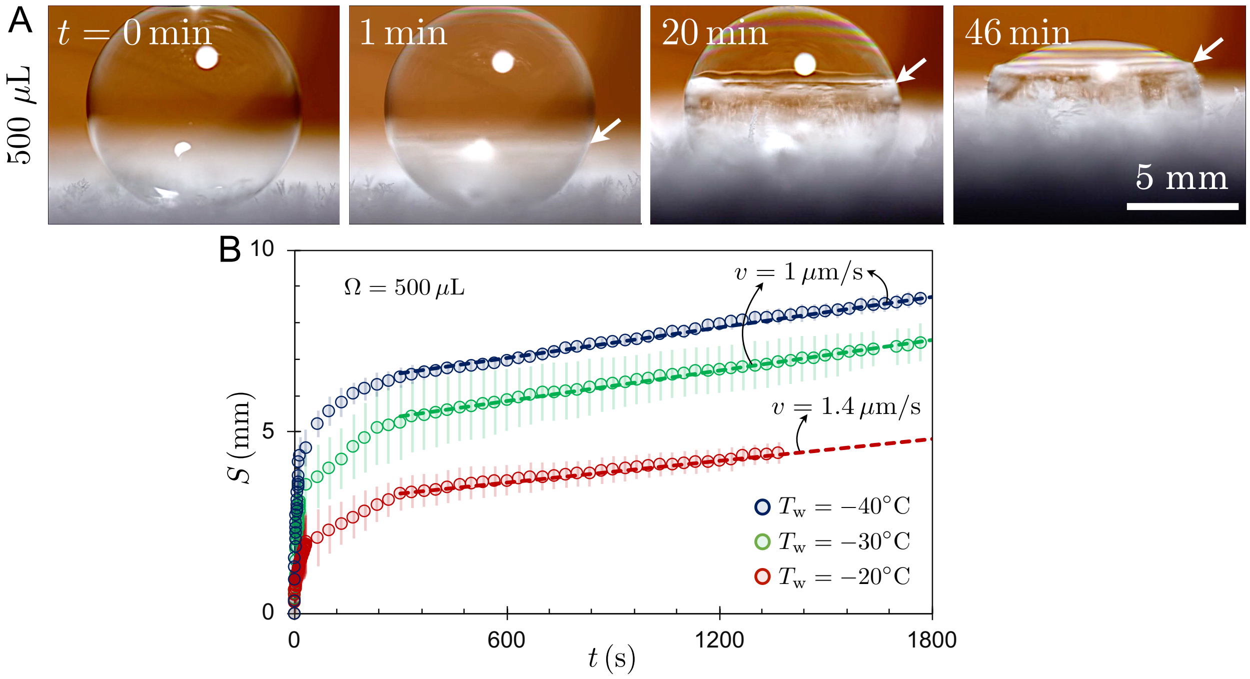

Supplementary Fig. 11: Effects of frost growth on bubble freezing. (A) For bubbles deposited on a frosted substrate in a room temperature environment (1st frame), the frost thickness grew over time. This served to gradually translate the thermal boundary layer, ζ, upward with respect to the Peltier and extend the maximal height of the bubble's freeze front. However, the progression and eventual halting of the freeze front itself was completed within 1 min (2nd frame), such that it was not directly affected by the frost growth aside from subsequently being translated (3rd and 4th frames). (B) The velocity of out-of-plane frost growth from the surface was modeled as $v \sim D(c_\infty - c_i)/\zeta$, where D is the diffusivity of water vapor in air, and c_∞ and c_i are the ambient vapor concentration and saturation vapor concentration over ice, respectively. For typical values of $D \sim 10^{-5}$ m^2/s, $c_\infty - c_i \sim 10^{-3}$ kg/m^3, and $\zeta \sim 10^{-2}$ m, the frost growth velocity is calculated as $v \sim 1$ μm/s which agrees with the experimental growth rate (dotted lines). Again, it is clear that the growth and halting of the freeze front is completed within tens of seconds (large initial slope), prior to being translated upward due to frost growth.

Supplementary Fig. 12

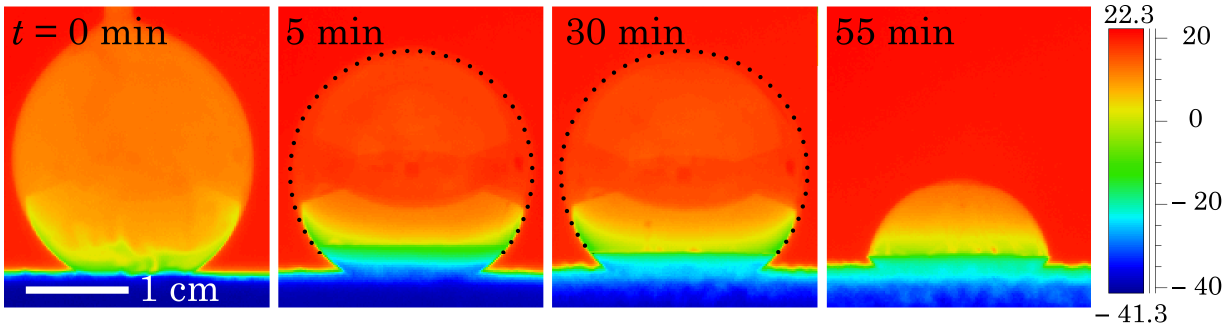

Supplementary Fig. 12: Thermographic images of freezing bubbles in a room temperature environment. A $\Omega = 10\,\text{mL}$ bubble was deposited on a frosted substrate of temperature $T_\text{w} = -40 \pm 1$ °C at a room temperature with $T_\infty = 23.3 \pm 1$ °C and $RH = 23 \pm 1.5\%$. The emissivity coefficient of ice was calibrated to $\epsilon = 0.98$. For better visualization of the periphery of the bubble (bubble-air interface), black dotted circles are drawn. While the latent heat did locally increase the temperature at the contact line, this did not generate strong Marangoni flows (as with the isothermal experiments) because the upper portion of the bubble was quite warm. The freeze front halted after $t_f \sim 10\,\text{s}$; the slight increase in the height of the freeze front over long time scales was simply due to frost growth on the substrate (see Supplementary Fig. 11). In the final frame, the liquid dome atop the bubble collapsed, which is discussed in Figure 6 of the main text.

Supplementary Fig. 13

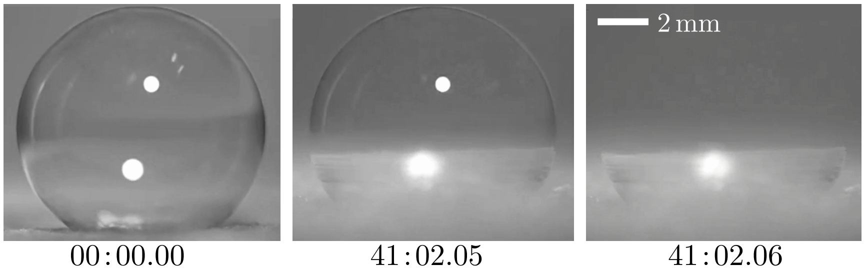

Supplementary Fig. 13: Bursting of a partially-frozen bubble. A $\Omega = 500$ μL bubble was deposited on a frosted substrate of temperature $T_{\mathrm{w}} = -30 \pm 1$ °C at a room temperature with $T_{\infty} = 23.8 \pm 1$ °C and $RH = 43.1\%$. The liquid dome atop the bubble was stable for 41 min and 2.05 sec (second frame). However, unlike the liquid collapse mechanism which is discussed in Figure 6 of the main text, the liquid dome atop the bubble pops immediately. The time lapse between the second and third frame is 10 ms.

Supplementary Fig. 14

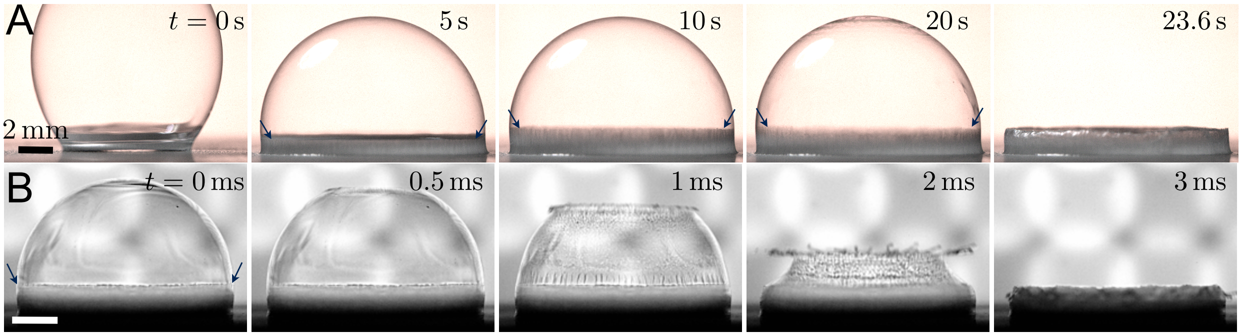

Supplementary Fig. 14: Control experiments using a pure surfactant. (A) Freezing of a $\Omega = 500\ \mu\text{L}$ bubble that was made using a solution containing 1% SDS, rather than dish soap, as the surfactant. The bubble was deposited on an icy substrate with a temperature of $T_\text{w} = -20 \pm 1\,^\circ\text{C}$ in an environment of $T_\infty = 22.1\,^\circ\text{C}$ and $RH = 13.7\%$. Arrows show the location of the freeze front. Bubbles with 1% SDS mostly burst within $\mathcal{O}(10\,\text{s})$. Drainage induced thinning of the bubble from top was responsible for the bursting of the bubble. (B) High-speed imaging showing the rapid bursting of a bubble at the end of its lifetime.

Supplementary Note 1: Computational Method

Thanks to the axisymmetric nature of a bubble, computations were performed in 2D as shown in Supplementary Fig. 8A. The 5 cm × 7.5 cm rectangular Peltier stage was approximated as a circular stage of radius $r_p = 3$ cm denoted by ob in Supplementary Fig. 8A. The bubble was placed at the center of the circular stage. The top edges around the Pelter stage were treated as rounded corners (ab) of radius $r_0 = 0.5$ cm, in order to avoid recirculations that may adversely affect convergence. A weakly compressible fluid flow is considered for the air outside the bubble; the velocity inside of the bubble is set to zero at all times. The no-slip condition was applied on the bubble surface (ta), Peltier stage (ab), and the transition corner (bc). The pressure at the upper corner of the domain space farthest from the Peltier (point f) was set to an atmosphere ($P_{\mathrm{atm}} = 1$ atm). Heat transfer was considered in the whole computational domain including the bubble. The bubble surface was defined as a sharp interface on which the temperature and heat flux are both continuous. The flow and heat transfer within the bubble film were neglected. The temperature at the Peltier stage (ob) was set to T_{w} while the temperature of the outer domain boundaries (de, ef, and fg) was set to T_∞. An adiabatic boundary condition was adopted on the rounded corner bc and vertical wall cd. The boundary go is the axis of axisymmetry where symmetry conditions are applied. Initially, the velocity was set to zero and the temperature was set to T_∞ inside of the whole domain. To obtain the solution at steady-state, each simulation was run for a flow time of 1,000 s. All computations were carried out using the laminar flow and heat transfer modules of COMSOL Multiphysics 5.3a.

The governing equations include the momentum equation

$$\rho\frac{\partial \mathbf{u}}{\partial t} + \rho(\mathbf{u}\cdot\nabla)\mathbf{u} = \nabla\cdot\left[-p\mathbf{I} + \mu\left(\nabla\mathbf{u} + (\nabla\mathbf{u})^T\right) - \frac{2}{3}\mu(\nabla\cdot\mathbf{u})\mathbf{I}\right] + \rho\mathbf{g}, \tag{1}$$

the continuity equation

$$\frac{\partial\rho}{\partial t} + \nabla\cdot(\rho\mathbf{u}) = 0, \tag{2}$$

and the heat equation

$$\rho C_{\mathrm{p}}\frac{\partial T}{\partial t} + \rho C_{\mathrm{p}}\mathbf{u}\cdot\nabla T = \nabla\cdot(k\nabla T). \tag{3}$$

Here $\mathbf{u}$ is the velocity, ρ is the density, p is the pressure, T is the temperature, and $\mathbf{g}$ is the gravitational acceleration. The equation of state and material parameters of the air, including the specific heat under constant pressure C_{p}, thermal conductivity k, and dynamic viscosity μ, are directly obtained from the built-in material library of COMSOL. Air convection/circulation inside the bubble has not been considered as the bubble surface is assumed to be immobile. In this case, the natural convection outside the bubble does not cause air motion inside the bubble. Moreover, the film thickness ($\sim 1\,\mu$m) is too small to elicit any appreciable thermal resistance across the film. Rather, a sharp interface used and the temperature is assumed continuous across the interface.
