# Supplementary material for: How soap bubbles freeze
Source: Nat Commun. 2019 Jun 18;10:2531. doi: 10.1038/s41467-019-10021-6 (PMC6582157; doi:10.1038/s41467-019-10021-6)
Supplement: Supplementary file 3 — Description of Additional Supplementary Files [file 41467_2019_10021_MOESM3_ESM.pdf]

Description of Additional Supplementary Files

Supplementary Movie 1: Freezing of Ω = 10 mL bubbles in a walk-in freezer with a temperature of $T_\infty \approx T_\mathrm{w} = -18.5$ °C and a relative humidity of RH = 60 ± 5%. It is observed that the Marangoni flow detached ice particles growing at the freeze front and carried them up the bubble.

Supplementary Movie 2: Freezing of bubbles deposited on a surface chilled to $T_\mathrm{w} = -40 \pm 1$ °C. The first portion of the video depicts the partial freezing of a larger bubble, followed by the collapse of the unfrozen liquid dome, while the second portion of the video shows the complete freezing of a smaller bubble. Ambient conditions are a room temperature of $T_\infty =$ 23.6 °C and a relative humidity of RH = 65.2 %.

Supplementary Movie 3: Freezing of Ω = 500 μL bubbles deposited on a Peltier stage cooled to a variety of surface temperatures. The critical height at which a freeze front comes to a halt is shown to increase with decreasing surface temperature. Ambient conditions are a room temperature of T_∞ = 23.5 ± 0.2% and a relative humidity of RH = 53.7 ± 0.4%.
